# Comparative study on growth traits and ions regulation of zoysiagrasses under varied salinity treatments

**DOI:** 10.1515/biol-2021-0079

**Published:** 2021-08-09

**Authors:** Zhenming Zhang, Huaguang Hu

**Affiliations:** Department of Bioscience of School of Marine and Bioengineering, Yancheng Teachers University, Yancheng 224007, China; Jiangsu Key Laboratory for Bioresources of Saline Soils, Yancheng Teachers University, Yancheng 224007, China

**Keywords:** growth traits, Na^+^ and K^+^ concentrations, salinity stress, zoysiagrass

## Abstract

Salt stress affects plant physiology, development, and growth. This research investigated varied salinity levels on growth traits and ions accumulation of four zoysiagrasses and aimed to identify phenotypic traits associated with variability in salinity tolerance. In this study, “S001” zoysiagrass (*Zoysia sinica*), “Diamond” zoysiagrass (*Zoysia matrella*), “J026” zoysiagrass (*Zoysia japonica*), and “M001” zoysiagrass (*Zoysia macrostachya*) were grown in plastic pots and exposed to 1/2 Hoagland nutrient solution amended with different amounts of NaCl for 120 days. At the end of the experiment, growth traits and ion contents were determined. The results showed that the salt-tolerance of four zoysiagrasses ranked as “M001” > “Diamond” > “J026” > “S001” according to percent green leaf canopy area (GLCA) after 120 days of salinity treatment. Although dry leaf weight, leaf length/width, and shoot height were significantly decreased by salinity treatments for all turfgrasses, the salt-tolerant species had a smaller drop. Besides, ions secretion capacity and Na^+^ concentration in leaf and root increased, but K^+^ concentration together with leaf and root K^+^/Na^+^ ratios decreased with the increasing concentration of the salinity. However, the salt-tolerant species exhibited strong K^+^ absorption and transportation ability and a high salt secretion capacity. The results indicated that growth traits and ions regulation were related to variability in tolerance of diverse zoysiagrasses to salt stress.

## 1 Introduction

Salt stress is considered as one of the environmental stresses affecting plant growth and development in some areas [1,2,3]. Salt stress causes plant’s physiological drought, ion toxicity, water and nutrient deficiency, and limits plant growth [4]. Severe salt stress can even result in plant death [5,6,7]. During turf establishment and maintenance, the use of saline and non-potable water leads to soil salinization. Additionally, high salinity content in the soil of coastal regions also increases the difficulty of turf establishment and maintenance. Growing salt-tolerant turfgrasses has been recognized as an effective way to handle this problem [8,9,10]. *Zoysia* Willd. plants are warm-season turfgrasses, which can be used extensively for varied turf establishment. Some zoysiagrasses exhibit strong resistance to salinity [11,12], which are ranked as halophytes, such as *Zoysia matrella*, *Zoysia japonica,* and *Zoysia macrostachya* [13]. These turfgrasses are promising to be used in turf establishment in salinized soil and coastal regions.

So far, many researchers have studied the physiological and growth responses of warm-season turfgrasses under salt stress. Leaf water content, K^+^ concentration, and osmotic potential were reduced under salt stress [14,15,16], while Na^+^ and Cl^‒^ concentrations, osmotic adjustment substances such as free proline, sugar and betaine, salt secretion capacity, and anti-oxidation enzymes activity such as peroxidase, catalase, and superoxide dismutase increased [17,18,19]. In terms of growth traits, salt stress inhibits cell elongation and decreases shoot biomass, leaf length, and width [20,21]. Some halophytic turfgrasses’ root growth was enhanced under low salinity treatment, while root growth was decreased when salinity level further increased [22,23]. These studies are very important to understand the effects of salt stress on turfgrasses, but these experiments are conducted in a short time, and little is known about the long-term effect of salt stress on growth traits and ions regulation of zoysiagrasses.

We carried out the experiment to study the responses of four zoysiagrasses to long-term salinity treatment, and the major objectives were to determine the growth traits and ions accumulation of four zoysiagrasses in the growing season with NaCl concentration ranging from 0 to 400 mM and to identify salt tolerance-related phenotypic traits.

## 2 Materials and methods

### 2.1 Plants growth

This study employed four zoysiagrasses, including “S001,” “Diamond,” “J026,” and “M001.” “S001,” the non-halophyte, was collected at the Jiaozhou Bay of Shandong Province, while the other three turfgrasses were halophytes, among which “Diamond” was a commercial turfgrass, and “J026” and “M001” were collected at Qingdao of Shandong Province and Yancheng of Jiangsu Province, respectively. On April 1st, 2019, stolons of four turfgrasses were collected from the biological garden of Yancheng Teachers University (33°21ʹN latitude and 120°09ʹE longitude) and were cut into stems (10–15 cm in length) with 6 sections. Then, the stems were planted in a plastic pot (15 cm in height and 10 cm in diameter) filled with river sand. Each zoysiagrass was planted into 15 pots, including 3 pots used as controls and 12 pots used as treatments. These pots were suspended in turnover boxes filled with 50 L  of l/2 Hoagland nutrient solution through the foam board. The turnover boxes were placed in the greenhouse and aerated with an air pump every day, the nutrient solution was replaced, and the plants were clipped once every week. The water level in turnover boxes was checked every two days, and water was supplied to make sure that the pot bottom always contacted the liquid level. Meanwhile, the pH of the nutrient solution was kept at 5.5–6.0.

### 2.2 Salinity treatment

Salt stress was conducted by the addition of NaCl to the l/2 Hoagland nutrient solution from June 1st, 2019, until the desired salinities of 100, 200, 300, and 400 mM were obtained, with 0 mM NaCl as control. To avoid the osmotic shock, salinity was incrementally increased by 50 mM every day. Salt stress lasted for 120 days. This study was repeated at the same period in 2020.

### 2.3 Data collection

To determine salt secretion capacity, salt crystals on the leaves’ surface were washed with deionized water on a sunny day after one week of salinity treatment. One week later, we cut ten mature leaves, put them at once into the centrifuged tube containing 5 mL of deionized water, and shook for 30 s to completely dissolve all leaf surface salt crystals into water. Later, we dumped the eluent to the other centrifuge tube for determining K^+^ and Na^+^ levels using the flame photometer (AA3600F, Corning, London, UK).

At the end of the experiment, ten leaves from every pot were cut and washed with deionized water, water on leaf surface was wiped and the fresh weight (FW) of leaf was measured at once. Afterwards, the leaves were transferred to Petri dishes containing deionized water and kept in dark for 4 h, so as to restore the turgidity. Subsequently, we eliminated excessive water on leaf surface to measure the turgid weight (TW), and the leaves were then dried at a temperature of 105°C for 30 min and then at 80°C for 48 h to measure their dry weight (DW). Subsequently, relative water content (RWC) was calculated according to the formula below.

\text{RWC} \% =(\text{FW}-\text{DW})/(\text{TW}-\text{DW})\times 100 \% \text{.}]

Percent green leaf canopy area (GLCA) of each pot was visually estimated. Shoot height, dry leaf weight, leaf length, and leaf width were determined with ten replications per pot. Then, both leaves and roots were ground into fine powders in the context of liquid nitrogen, and digested by 0.5% of HNO_3_ solution for ion extraction. Subsequently, the content of Na^+^ and K^+^ were measured using the flame photometer mentioned earlier.

### 2.4 Experimental design and data analysis

The experiment was performed under complete randomization condition, and each salinity treatment was repeated three times. Differences in data between 2019 and 2020 were not statistically significant, and as a result, data collected at the sampling dates in these two years were combined and the means were used for statistical analysis. The Excel software (Version 2010, MS Corporation, Redmond, USA) was used to draw histograms. The one-way ANOVA was utilized for data analysis by employing the SAS software (Version 9.4, SAS Institute, Cary, NC, USA). Meanwhile, the Duncan’s multiple range test was performed to compare the averages, and the significance level was set as *p* ≤ 0.05.

## 3 Results

### 3.1 GLCA percent

**Figure 1:**
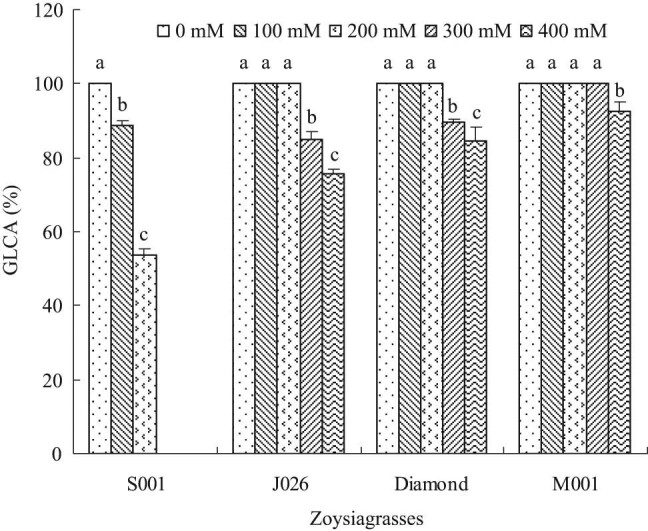
GLCA percent of four zoysiagrasses under different salinity levels. Means followed by the same superscript lowercase letters are not significant difference at *p* < 0.05 level.

The GLCA percent of four turfgrasses showed the significant difference under salt stress (Figure 1). Specifically, “J026” and “Diamond” kept 100% GLCA when the salinity level was 200 mM. However, their GLCA percent decreased significantly at 300 and 400 mM salinity levels. “J026” achieved 85.00 and 75.67% GLCA at 300 and 400 mM salinity levels, respectively, whereas, the GLCA percent of “Diamond” were 89.67 and 84.33%, respectively. “M001” still maintained 100% GLCA at the salinity level of as high as 300 mM, but its GLCA percent significantly decreased to 92.67% at 400 mM salinity level. As for “S001,” the GLCA percent decreased gradually as the salinity level increased, it died at the 300 mM salinity level on the 20th day of the experiment, and on the 14th day under 400 mM salinity treatment. It was concluded based on the above findings that, “M001” was a high salt-tolerant species, whereas “J026” and “Diamond” were the moderate salt-tolerant species, and “S001” was a salt-sensitive species.

### 3.2 Shoot height

**Figure 2:**
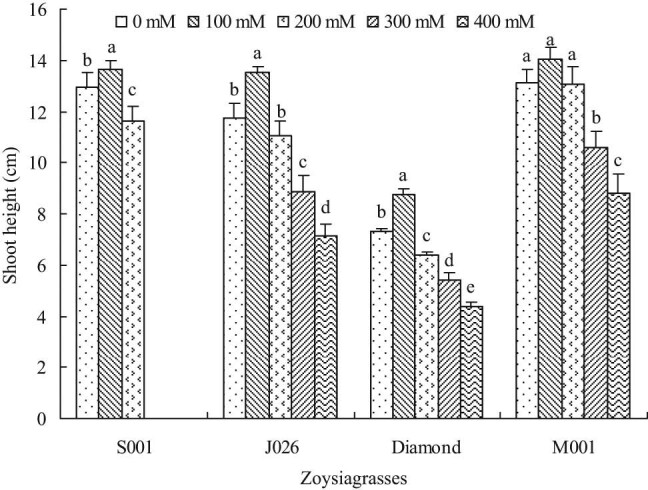
Shoot height of four zoysiagrasses under different salinity levels. Means followed by the same superscript lowercase letters are not significant difference at *p* < 0.05 level.

The shoot height of four turfgrasses slightly increased at the salinity level of 100 mM, but it reduced gradually as the salinity concentration increased to other levels (Figure 2). Such results indicated that shoot growth was promoted at low salinity concentration (≦100 mM) but inhibited at high salinity concentration (>100 mM). Typically, the shoot height was not significantly affected at the salinity level of 200 mM, but it significantly decreased at the salinity levels of 300 and 400 mM for “J026” and “M001.” Besides, the shoot height of “J026” and “M001” decreased by 39.39 and 32.83%, respectively, at 400 mM salinity level compared with the control. The shoot height of “S001” and “Diamond” decreased significantly under 200 mM salinity treatment, while that of “S001” remained constant under 300 and 400 mM salinity treatments due to death, and that of “Diamond” showed a gradual decline at 300 and 400 mM salinity levels.

### 3.3 Leaf water content

**Figure 3:**
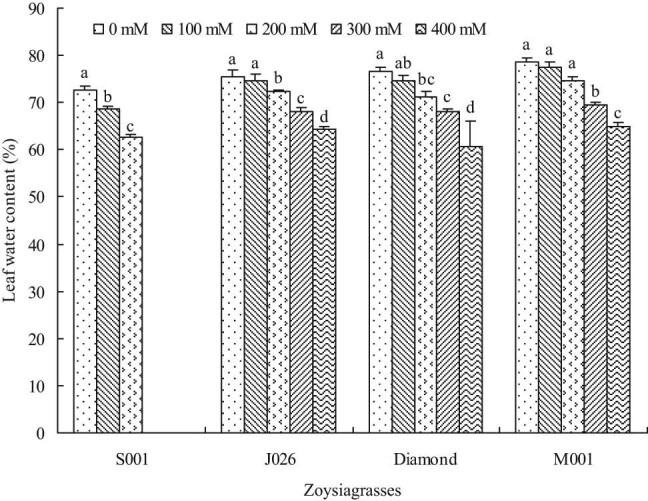
Leaf water content of four zoysiagrasses under different salinity levels. Means followed by the same superscript lowercase letters are not significant difference at *p* < 0.05 level.

After 120 days of salinity treatment, the leaf water content was not affected at the 100 mM salinity level in all turfgrasses except for “S001” with 5.23% decrease. At the 200 mM salinity level, leaf water content significantly declined in all turfgrasses except for “M001” with largely unaffected leaf water content. The leaf water contents of “J026,” “Diamond,” and “M001” significantly decreased at 300 and 400 mM salinity levels; typically, under 400 mM NaCl treatment, the leaf water content of the turfgrasses “J026,” “Diamond,” and “M001” decreased by 14.91, 20.70, and 17.18%, respectively, compared with control (Figure 3).

### 3.4 Leaf length, width, and weight

**Figure 4:**
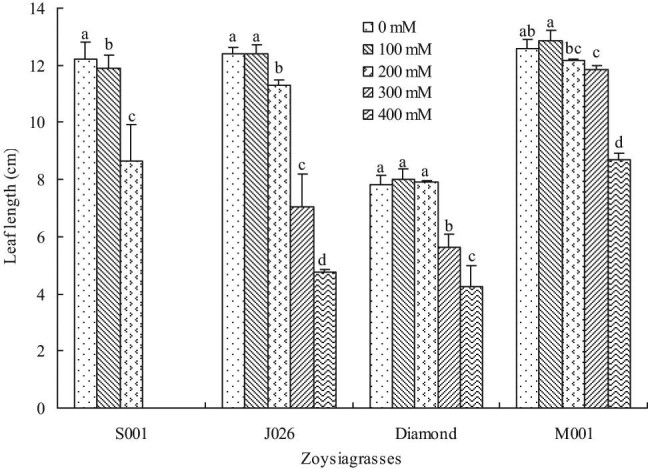
Leaf length of four zoysiagrasses under different salinity levels. Means followed by the same superscript lowercase letters are not significant difference at *p* < 0.05 level.

**Figure 5:**
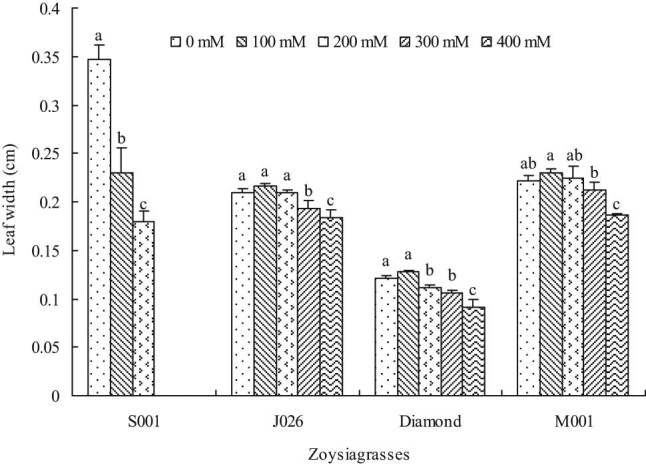
Leaf width of four zoysiagrasses under different salinity levels. Means followed by the same superscript lowercase letters are not significant difference at *p* < 0.05 level.

**Figure 6:**
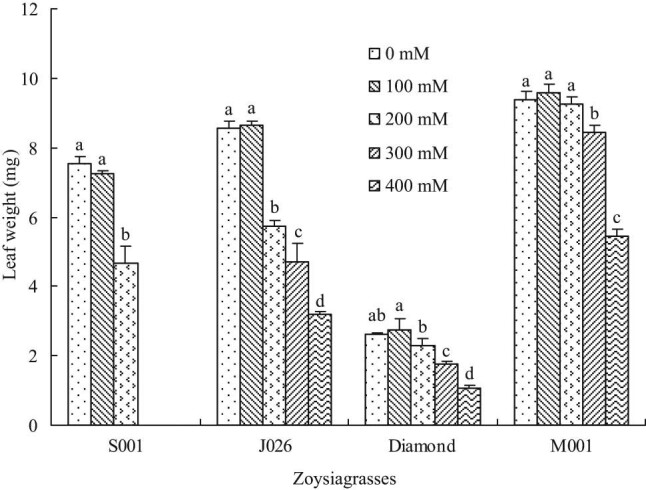
Leaf weight of four zoysiagrasses under different salinity levels. Means followed by the same superscript lowercase letters are not significant difference at *p* < 0.05 level.

Leaf length was significantly reduced in “S001” under 100 mM salinity treatment, but there was no significant change in the remaining three species. Besides, the leaf length of all turfgrasses significantly reduced at 200 mM salinity concentration compared with control, except for “Diamond.” Moreover, the leaf length of “J026,” “Diamond,” and “M001” significantly decreased at 300 and 400 mM salinity levels, and the leaf length decreased by 61.63, 45.32, and 30.87%, respectively, at 400 mM salinity level compared with control. The results showed that “M001” had a greater growth capacity at higher salinity concentration (Figure 4). As a key indicator of turf texture, leaf width showed a similar variation pattern to that of leaf length. Different from leaf length, 200 mM NaCl treatment did not affect the leaf width of “J026” and “M001,” but that of “Diamond” significantly decreased. Compared with control, the leaf width decreased by 12.85% in “J026,” 13.81% in “Diamond,” and 15.00% in “M001” under 400 mM NaCl treatment (Figure 5). In addition, the leaf weight was not significantly affected in all turfgrasses under 100 mM salinity treatment and in “M001” at 200 mM salinity level; however, the leaf weight significantly reduced under other salinity treatments compared with control (Figure 6).

### 3.5 Ions secretion capacity

The secretion capacity of K^+^ and Na^+^ ions increased significantly in all turfgrasses with the increase in salinity concentration. Moreover, “M001” exhibited the strongest secretion capacity, in which the K^+^ and Na^+^ ions secretion efficiency increased by 97.15–350.53% and 899.15–4692.22%, respectively, at the 100–400 mM salinity levels relative to control. Meanwhile, the Na^+^/K^+^ ratio in “M001” was the highest at each salinity level compared with those in the other three turfgrasses (Table 1).

**Table 1:** Salt secretion capacity of four turfgrasses after 120 days of salt stress (mmol Kg^−1^)

Salinity concentration	S001			J026			Diamond			M001		
	K^+^	Na^+^	Na^+^/K^+^	K^+^	Na^+^	Na^+^/K^+^	K^+^	Na^+^	Na^+^/K^+^	K^+^	Na^+^	Na^+^/K^+^
0	3.60c	5.18c	1.44	4.30e	4.85e	1.13	5.38e	5.36e	1.00	5.64e	5.91e	1.05
100	9.02b	22.38b	2.48	7.34d	23.79d	3.24	10.29d	36.73d	3.57	11.09d	59.05d	5.32
200	16.30a	53.72a	3.30	11.44c	44.63c	3.90	14.89c	65.78c	4.42	14.68c	80.39c	5.48
300	0.00	0	0.00	16.34b	71.99b	4.41	21.31b	93.0b	4.37	21.08b	135.85b	6.44
400	0.00	0	0.00	18.14a	103.40a	5.70	24.11a	105.09a	4.36	25.41a	283.22a	11.15

Note: Means followed by the different letters in the same column are significant difference (*p <* 0.05).

### 3.6 Uptake of Na^+^ and K^+^

An increasing amount of Na^+^ was absorbed in root as the salinity concentrations elevated, which resulted in the gradual increase in Na^+^ concentrations in all turfgrasses. Na^+^ concentrations in “S001” and “Diamond” significantly increased at each salinity level; to be specific, it increased by 3.04 folds in “S001” at the 200 mM salinity level and by 8.80 folds in “Diamond” at the 400 mM salinity level, compared with control. As for “J026” and “M001,” Na^+^ concentrations also significantly increased, but no significant differences were detected between the 100 and 200 mM salinity levels. Additionally, Na^+^ concentrations elevated by 9.21 folds in “J026” and by 7.15 folds in “M001,” at the 400 mM salinity level compared with control. Na^+^ transportation from root to leaf increased, as a result, the leaf Na^+^ concentration significantly increased at every salinity level except for “J026,” with no significant differences between the 300 and 400 mM salinity levels. As the salinity level further increased, the leaf Na^+^/Root Na^+^ ratio first increased and then decreased in all the turfgrasses except for “S001” that showed continuous reduction (Table 2).

**Table 2:** Leaf and root Na^+^ and K^+^ concentrations of four turfgrasses after 120 days of salt stress

Species	Salinity concentrations	Leaf Na^+^	Root Na^+^	Leaf Na^+^/root Na^+^	Leaf K^+^	Root K^+^	Leaf K^+^/Root K^+^	Leaf K^+^/Na^+^	Root K^+^/Na^+^
S001	0	87.31c	58.48c	1.49a	257.0a	124.65a	2.064a	2.95a	2.13a
	100	136.52b	115.81b	1.17b	168.36b	107.60b	1.56b	1.24b	0.93b
	200	181.31a	178.12a	1.02c	95.30c	84.14c	1.14c	0.53c	0.47c
J026	0	87.97d	49.46d	1.78b	457.06a	165.40a	2.76b	5.19a	3.35a
	100	253.67c	105.15c	2.43a	325.36b	140.31b	2.32d	1.29b	1.34b
	200	310.43b	117.41c	2.66a	292.18c	111.19c	2.63cd	0.95c	0.95c
	300	392.88a	234.3b	1.68b	270.45c	87.77d	3.08a	0.69cd	0.38d
	400	429.39a	455.71a	0.94c	195.11d	81.76d	2.38bc	0.46d	0.18d
Diamond	0	83.63e	48.88e	1.71c	540.52a	170.11a	3.18a	6.47a	3.48a
	100	326.20d	121.61d	2.69a	370.42b	139.18b	2.66b	1.14b	1.14b
	200	423.88c	209.30c	2.02b	232.17c	118.52c	1.95c	0.55c	0.57c
	300	477.69b	336.79b	1.42cd	213.90d	93.23d	2.29bc	0.45c	0.28d
	400	531.19a	430.34a	1.23d	186.03d	82.10d	2.26bc	0.35c	0.19d
M001	0	79.31e	45.29d	1.75b	557.01a	174.65a	3.19c	7.03a	3.86a
	100	176.52d	104.78c	1.68b	568.36a	184.93a	3.07c	3.23b	1.77b
	200	255.97c	118.12c	2.17a	586.64a	182.47a	3.22c	2.30c	1.55c
	300	317.45b	217.34b	1.46c	522.64b	135.03b	3.87b	1.6d	0.62d
	400	383.21a	323.86a	1.18d	483.51c	106.07b	4.56a	1.26e	0.33e

Note: Means of each turfgrass followed by the different letters in the same column are significant difference (*p* < 0.05).

K^+^ concentrations in root and leaf showed a different variation trend from that of Na^+^. K^+^ concentration in root decreased gradually as the salinity level increased in all turfgrasses compared with control. Besides, K^+^ concentration in root significantly decreased at the 100 and 200 mM NaCl levels except for “M001,” which showed no significant change between the 100 and 200 mM and between the 300 and 400 mM salinity levels. In the meantime, at the 400 mM salinity level, K^+^ concentration in “M001” was higher than those in “J026,” “S001,” and “Diamond.” Furthermore, salt stress inhibited K^+^ transportation from root to leaf, therefore, the leaf K^+^ concentration decreased at every salinity level, but it was still higher than that in root, and the leaf K^+^/Root K^+^ ratio was >1. At the 400 mM salinity level, K^+^ concentration in “M001” was 2.48 times as high as that in “J026” and 2.60 times as that in “Diamond.”.

The root and leaf K^+^/Na^+^ ratios showed a gradual declining trend as the salinity level continued to increase. It indicated that salt stress promoted the absorption and transportation of Na^+^ but inhibited those of K^+^, which led to the imbalance between K^+^ and Na^+^ in cells, but the root and leaf K^+^/Na^+^ ratios in “M001” were greater than those in the other three turfgrasses.

## 4 Discussion

Based on the percent leaf firing data, Marcum Kenneth et al. [24] and Qian et al. [12] reported that the “Diamond” zoysiagrass showed the highest salinity tolerance in their experiments. Based on the GLCA results, Chen et al. [25] suggested that “Diamond” was the salt-tolerant species, whereas “Z080” zoysiagrass (*Z. japonica*) was the salt-sensitive species. In our research, “Diamond” zoysiagrass was the moderate salt-tolerant species, which supported the previous research.

RWC is an important physiological index to evaluate the salt tolerance of plant. According to previous research, the RWC of *Cynodon dactylon* [14] and *Agropyron cristatum* [26] decreased under salt stress. In this experiment, we found that the RWC of four zoysiagrasses decreased after 120 days of salt stress, but “M001” still kept a higher RWC at high salinity concentration, which also indirectly proved that “M001” exhibited stronger salt-tolerant capacity.

As suggested by Dudeck and Peacock [27], Lee et al. [28,29], and Pompeiano et al. [17], salt stress obstructed plant growth and resulted in marked biomass loss. According to our experimental data, the dry leaf weight of four turfgrasses were not markedly changed at low salinity levels, but were significantly lost at high salinity treatment. Salinity stress suppressed the elongation of cells and inhibits the growth of leaves [20,21]. In Hu and Schmidhalter’s study [30], the *Triticum aestivum* leaves were reduced in width and length after exposure to salinity stress. For the present study, “M001” showed the highest salt tolerance but still had the greatest shoot height among all turfgrasses under high salinity treatment. Besides, its length, width, and dry leaf weight suffered the least reduction. Based on such results, the leaf growth traits and shoot height were the vital parameters affecting the tolerance to salinity.

It was reported by Marcum and Murdoch [31] that salt glands existed on zoysiagrasses leaf surface, which contributed to the selective secretion of Na^+^ and Cl^−^, thus reducing the salt stress-induced damage. Moreover, the salt tolerance is reported to be significantly and positively correlated with the salt secretion ability of zoysiagrasses [9,31]. In this study, zoysiagrasses were found to secrete K^+^ and Na^+^ ions, among which “M001” was the most efficient in secreting ions.

Salt stress affects ion absorption and transportation in plants [32]. K^+^ and Na^+^ uptake or K^+^/Na^+^ ratio were related to the salt tolerance in some plant species [22,33]. For instance, wheat with tolerant genotypes keeps the K^+^ uptake high, Na^+^ absorption low, and thereby the leaf K^+^/Na^+^ ratio high [32]. As revealed by this study, “M001,” the salt-tolerant species, showed high K^+^ uptake and low Na^+^ accumulation in root and leaf at high salinity levels, which conformed to other studies [15,25,34]. Such findings indicated that it was beneficial to promote K^+^ uptake and avoid excess Na^+^ accumulation to enhance the salt tolerance of “M001.” Potassium stands for one of the critical nutrient elements necessary during the growth and development of plants. Na^+^ almost has the same ionic radius as K^+^, which results in the competitive absorption of K^+^ with Na^+^ within cell membrane in the presence of salt stress [35]. In addition, the root and leaf K^+^/Na^+^ ratios are correlated with the salt tolerance of plant. As demonstrated by Guo et al. [15], seashore paspalum (*Paspalum vaginatum*), the salt-tolerant species, showed greater root and leaf K^+^/Na^+^ ratios. In this work, “M001” had greater root and leaf K^+^/Na^+^ ratios than that of the other three turfgrasses. Therefore, the capacity of K^+^ uptake and root-to-leaf Na^+^ transportation inhibition was suggested to be of vital significance to the salt tolerance in “M001.”

Taken together, the “M001,” “Diamond,” and “J026” can adapt their growth to salt stress for 120 days at every levels, and their GLCA percent still remained at above 70% under 400 mM NaCl treatment, demonstrating that they had stronger salt-tolerant capacity. However, the “S001” died on the 20th day under 300 mM NaCl treatment and on the 14th day at the 400 mM salinity level. According to the GLCA percent, after 120 days of salinity treatment, the salt tolerance of four turfgrasses followed the order of “M001” > “Diamond” > “J026” > “S001.” The growth traits and physiological indexes, such as leaf length and width, leaf weight, and RWC were reduced to varying degrees under salinity treatment. Compared with S001, the salt-tolerant zoysiagrasses in this experiment exhibited strong capacity for K^+^ absorption and transportation, high salt secretion capacity, high GLCA percent and shoot height, and low leaf growth reduction, all of which might contribute to the strong salt tolerance of these turfgrasses.
